# Apixaban concentration variability and relation to clinical outcomes in real-life patients with atrial fibrillation

**DOI:** 10.1038/s41598-021-93372-9

**Published:** 2021-07-06

**Authors:** Alenka Mavri, Nina Vene, Mojca Božič-Mijovski, Marko Miklič, Lisbeth Söderblom, Anton Pohanka, Rickard E. Malmström, Jovan Antovic

**Affiliations:** 1 Department of Vascular Diseases, University Medical Centre Ljubljana, Zaloška 2, 1000 Ljubljana, Slovenia; 2 Department of Coagulation Research, Institute for Molecular Medicine and Surgery, Karolinska Institutet, Stockholm, Sweden; 3 Division of Clinical Pharmacology, Department of Laboratory Medicine Huddinge, Karolinska Institutet and Clinical Pharmacology, Karolinska University Hospital, Stockholm, Sweden; 4 Department of Medicine Solna, Karolinska Institutet and Clinical Pharmacology, Karolinska University Hospital, Stockholm, Sweden; 5 Faculty of Medicine, University of Ljubljana, Ljubljana, Slovenia; 6 Department of Clinical Biochemistry, Faculty of Pharmacy, University of Ljubljana, Ljubljana, Slovenia

**Keywords:** Drug regulation, Cardiovascular diseases

## Abstract

In some clinical situations, measurements of anticoagulant effect of apixaban may be needed. We investigated the inter- and intra-individual apixaban variability in patients with atrial fibrillation and correlated these results with clinical outcome. We included 62 patients receiving either 5 mg (A5, n = 32) or 2.5 mg (A2.5, n = 30) apixaban twice-daily. We collected three trough and three peak blood samples 6–8 weeks apart. Apixaban concentration was measured by liquid chromatography-tandem mass-spectrometry (LC–MS/MS) and by anti-Xa. Patients on A2.5 were older, had lower creatinine clearance, higher CHA_2_DS_2_VASc (4.7 ± 1.0 vs. 3.4 ± 1.7) and lower trough (85 ± 39 vs. 117 ± 53 ng/mL) and peak (170 ± 56 vs. 256 ± 91 ng/mL) apixaban concentrations than patients on A5 (all *p* < 0.01). In patients on A5, LC–MS/MS showed a significant difference between through levels and between peak levels (*p* < 0.01). During apixaban treatment, 21 patients suffered bleeding (2 major). There was no association between bleeding and apixaban concentrations or variability. Four patients who suffered thromboembolic event had lower peak apixaban concentrations than patients without it (159 ± 13 vs. 238 ± 88 ng/mL, *p* = 0.05). We concluded, that there was a significant intra- and inter-individual variability in apixaban trough and peak concentrations. Neither variability nor apixaban concentrations were associated with clinical outcomes.

## Introduction

Apixaban is a direct, oral, reversible, and highly selective inhibitor of activated factor X (FXa) that inhibits free and clot-bound FXa, as well as prothrombinase activity, which inhibits clot growth. It has been approved for clinical use in several thromboembolic disorders, including reduction of stroke risk in non-valvular atrial fibrillation, thromboprophylaxis following hip or knee replacement surgery, treatment of deep vein thrombosis or pulmonary embolism, and prevention of recurrent deep vein thrombosis and pulmonary embolism^1^.

The ARISTOTLE trial demonstrated that fixed-dose unmonitored apixaban compared with dose-adjusted warfarin was associated with a similar rate of ischemic stroke and a reduction in haemorrhagic stroke and major bleeding. Apixaban was administered as 5 mg twice daily unless patients met two or more criteria for a reduced dose apixaban 2.5 mg twice daily (age ≥ 80 years, body weight ≤ 60 kg, and serum creatinine > 1.5 mg/dL). A systematic review and meta-analysis of 16 studies on apixaban in real-life found a similar effectiveness in reducing stroke and a better safety profile for apixaban when compared to warfarin^2^. The AVERROES pharmacokinetic substudy demonstrated considerable variability in anti-Xa levels in patients with atrial fibrillation receiving apixaban and found no association between apixaban trough anti-Xa levels and risk of both major bleeding and stroke^3^. The efficacy and safety of apixaban over a wide range of anti-Xa levels does not support routine laboratory monitoring. However, there are certain clinical instances in which measurement of anticoagulant activity may be desirable, such as bleeding complications or the occurrence of thrombosis during anticoagulant therapy, prior to major surgery to detect residual anticoagulant drug effect, in patients with renal impairment, extreme body weight, and suspected noncompliance or overdose. Yet, only "expected ranges" for apixaban levels have been reported, while therapeutic ranges have not been defined.

Apixaban concentration can be measured directly by liquid chromatography with tandem mass spectrometry (LC -MS/ MS), but the method is not widely available. Chromogenic anti-factor Xa assays calibrated with known concentrations of apixaban are suitable for quantifying a wide range of apixaban plasma concentrations^4,5^. Considerable inter-patient variability is observed in apixaban concentrations measured by both methods^3,5–7^, and scarce data on intra-individual variability are available^8,9^. There is at least tenfold variation in apixaban trough plasma concentration for both dosing^5^ and even greater, a 50-fold variation is observed at any time point from the last dosage^7^. In real-life atrial fibrillation patients variability is associated with factors such as sex, renal function and heart failure^10^. Therefore, the aim of this study was to evaluate the intra- and inter-individual plasma apixaban variability in patients with atrial fibrillation in routine care and to investigate the possible association between apixaban concentration and adverse events.

## Results

The characteristics of the patients are shown in Table 1. Patients on A2.5 were significantly older, had significantly lower body weight and creatinine clearance (CrCl), and higher CHA2DS2VASc and HAS -BLED scores than patients on A5. There were more females than males on A2.5, but their trough or peak apixaban concentrations did not differ significantly.

**Table 1:** Characteristics of the patients on apixaban 5 mg (A5) or 2.5 mg (A2.5) twice a day.

	All N = 62	A5 N = 32	A2.5 N = 30	A5 versus A2.5 p
Sex, female/male	37/25	16/16	21/9	0.18
Age (years)	78 ± 8	73 ± 8	83 ± 3	< 0.01
Body weight (kg)	75.6 ± 16	82 ± 15	69 ± 13	< 0.01
Creatinine (µmol/L)	89 ± 24	82 ± 18	98 ± 27	< 0.01
CrCl (mL/min)	63 ± 24	78 ± 23	46 ± 12	< 0.01
Arterial hypertension	53 (85.5)	27 (84.4)	26 (86.7)	0.86
Diabetes mellitus	10 (16.1)	6 (18.8)	4 (13.3)	0.82
Heart failure	17 (27.4)	5 (15.6)	12 (40.0)	0.06
Ischemic heart disease	15 (24.2)	8 (25.0)	7 (23.3)	0.89
Peripheral artery disease	3 (4.8)	1 (3.1)	2 (6.7)	0.95
Previous stroke or systemic embolism	10 (16.1)	4 (12.5)	6 (20.0)	0.65
CHA_2_DS_2_VASc score	4.0 ± 1.5	3.4 ± 1.7	4.7 ± 1.0	< 0.01
HAS-BLED score	1.1 ± 0.5	0.9 ± 0.6	1.2 ± 0.4	0.03

Average ± SD or number of cases (%) is given.

*CrCl* creatinine clearance estimated by the Cockcroft–Gault equation.

**Figure 1:**
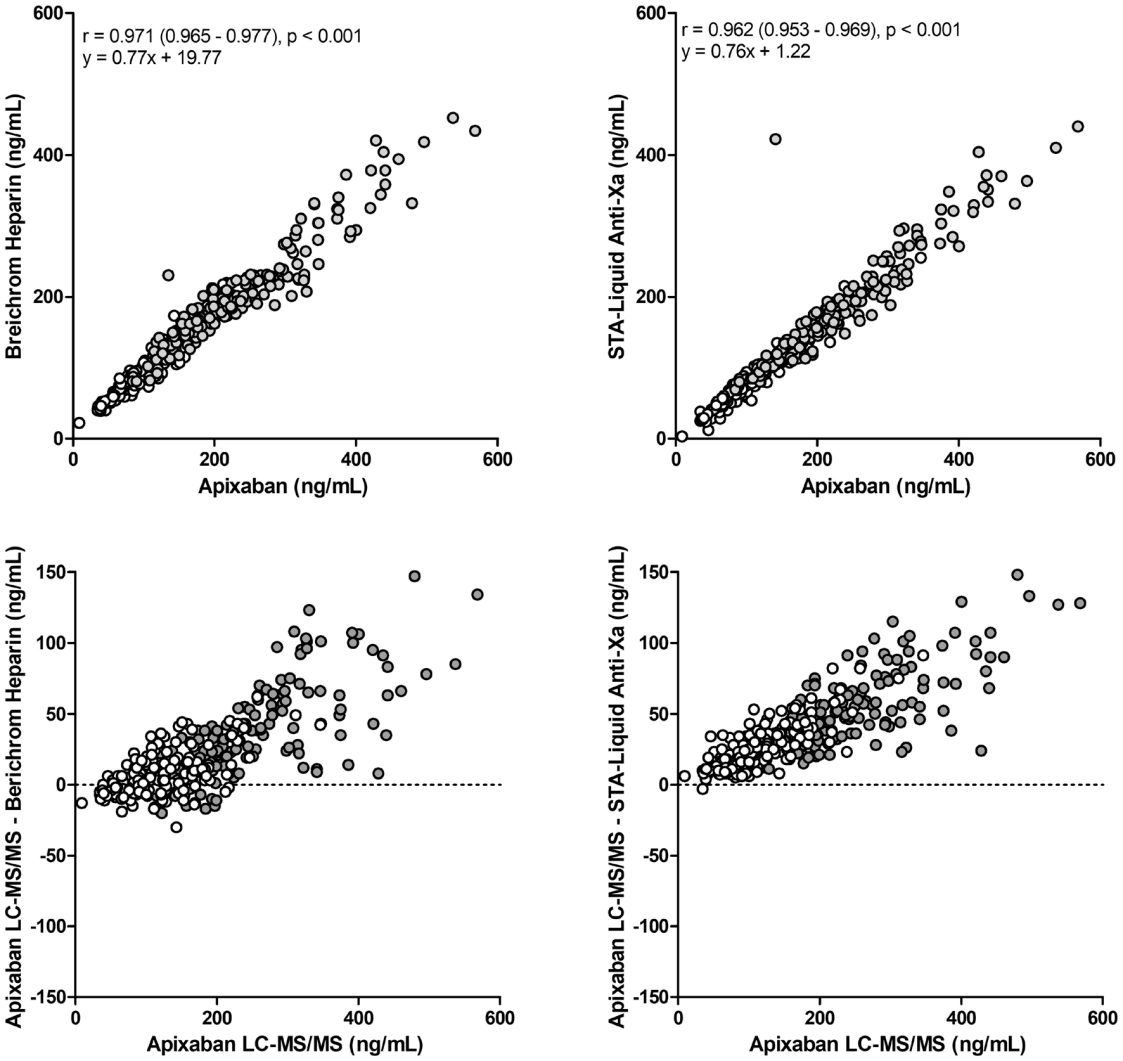
Correlations between apixaban plasma concentrations measured by liquid chromatography-tandem mass spectrometry (LC–MS/MS) and two anti-Xa assays, measured in two different laboratories (upper panel) and Bland–Altman plot (lower panel). Full circles at peak and empty circles at trough.

Apixaban concentration determined by LC–MS/MS showed significant correlation with the two different anti-Xa assays performed in two different laboratories (Fig. 1, upper panel), however, both methods underestimated apixaban concentration as shown by the Bland–Altman plot (Fig. 1, lower panel). There was no significant correlation between apixaban concentration and PT or APTT.

Table 2 shows apixaban concentrations determined by LC–MS/MS and anti-Xa coagulation assays in the trough and peak samples. In patients on A5, the comparison of repeated measurements showed significant differences in the three blood sample time points at the trough and peak measured by LC–MS/MS (ANOVA *p* ≤ 0.01) and at the peak measured by anti-Xa (ANOVA *p* ≤ 0.05). In patients on A2.5, the comparison of repeated measurements of trough and peak apixaban concentration showed no significant differences.

**Table 2:** Apixaban measurements in three peak and three trough samples in patients on apixaban 5 mg (A5) or 2.5 mg (A2.5) twice-daily with the corresponding intra-individual coefficient of variation (CV).

	Trough 1	Trough 2	Trough 3	ANOVA p	Average trough	Average CV (%)	Peak 1	Peak 2	Peak 3	ANOVA p	Average peak	Average CV (%)
**LC–MS/MS (ng/mL)**												
A5	141 (104–176) *(62*–*346)*	117 (82–146) *(51*–*229)*	108 (90–165) *(40*–*311)*	0.01	117 (93–161) *(63*–*291)*	19 ± 13	300 (225–382) *(125*–*537)*	232 (177–314) *(85*–*460)*	254 (177–346) *(90*–*568)*	0.002	256 (202–345) *(135*–*488)*	20 ± 14
A2.5	99** (67–125) *(9*–*188)*	84** (66–104) *(35*–*246)*	89* (65–109) *(35*–*212)*	0.07	85** (68–110) *(41*–*200)*	23 ± 17	183** (149–246) *(106*–*421)*	184** (131–208) *(86–386)*	164** (143–200) *(84–316)*	0.48	170** (145–216) *(109*–*374)*	20 ± 9
**Berichrom Heparin anti-Xa (ng/mL)**												
A5	131 (83–158) *(56–304)*	115 (79–141) *(54–219)*	107 (85–157) *(46–262)*	0.60	115 (87–152) *(62*–*262)*	21 ± 15	227 (194–309) *(131*–*452)*	194 (116–246) *(87*–*420)*	215 (168–246) *(81*–*434)*	0.007	206** (178–269) *(126*–*396*)	17 ± 11
A2.5	87** (66–105) *(22*–*182)*	80* (66–96) *(42*–*227)*	85* (60–101) *(39*–*217)*	0.20	83** (64–104) *(46*–*206)*	18 ± 13	162** (135–197) *(92*–*378)*	167* (123–197) *(89–372)*	157** (144–182) *(81–294)﻿*	0.66	165 (133–184) *(105–348)﻿*	17 ± 9
**STA–Liquid anti-Xa (ng/mL)**												
A5	106 (67–127) *(28*–*255)*	98 (70–115) *(41*–*180)*	85 (73–135) *(29*–*236)*	0.90	93 (71–121) *(47*–*224)*	29 ± 17	213 (170–279) *(95*–*410)*	178 (149–261) *(78*–*404)*	215 (156–273) *(80*–*440)*	0.05	197** (154–274) *(109*–*388)*	17 ± 12
A2.5	73** (49–90) *(3*–*137)*	68** (52–84) *(24*–*195)*	72* (48–94) *(27*–*183)*	0.89	70** (53–93) *(27*–*171)*	30 ± 22	129** (113–174) *(80–329)*	143** (104–167) *(69–348)*	134** (118–179) *(69–422)*	0.94	138 (107–177) *(84–323)*	20 ± 16

Results are shown as median with first to third quartile (roman font) and minimum to maximum range (italic font).

**p < 0.01, *p < 0.05 for Mann–Whitney comparison between patients on A5 and A2.

There was no significant difference between the average intra-individual CV of the trough apixaban concentration and the CV of the peak apixaban concentration measured with LC–MS/MS and with Berichrom Heparin anti-Xa. However, the average intra-individual CV of the trough apixaban concentration measured with STA–Liquid anti-Xa was significantly higher than the CV of the peak apixaban concentration (29 ± 17 vs. 17 ± 12, *p* < 0.01 for patients on A5 and 30 ± 22 vs. 20 ± 16, *p* = 0.05 for patients on A2.5). Patients with the highest trough apixaban concentration intra-individual variability (with the CV above the upper quintile, N = 12) had lower body weight (68 ± 18 vs. 77 ± 15 kg), lower creatinine concentration (75 ± 19 vs. 92 ± 24 µmol/L) and lower average trough apixaban concentration (87 ± 26 vs. 121 ± 52 ng/mL) than those with the trough apixaban concentration CV below the upper quintile.

Apixaban concentration measured by LC–MS/MS was significantly lower in patients on A2.5 compared to patients on A5 for through (27%) and for peak (34%) values (Table 2). These differences were also evident with both anti-Xa assays. Only 9 (30%) patients on A2.5 fulfilled the manufacturer recommendation for the apixaban dose reduction. The other patients had only one criterion: age ≥ 80 years in 18 patients, body weight ≤ 60 kg in 2 patients). One patient received a reduced dose due to concomitant dual antiplatelet treatment. There was no difference in apixaban concentrations between patients who fulfilled criteria for reduced dose and those who did not (median with first to third quartile: 87 (72–113) vs. 85 (68–110) ng/mL, *p* = 0.69 for average trough levels and 214 (158–255) vs. 167 (145–200) ng/mL, *p* = 0.23 for average peak levels).

Twenty-one patients suffered bleeding during the treatment with apixaban (12 on A5 and 9 on A2.5). We documented 2 major bleeding (1 posttraumatic subdural haematoma and 1 intraocular bleeding) and 19 spontaneous minor bleeding (5 epistaxis, 8 mucosal and skin bleeding, 3 occult gastrointestinal, and 3 urologic bleeding). Among thromboembolic events one transient ischemic attack (on A5) and 3 acute myocardial infarctions (on A2.5) were recorded. No differences in the average trough or peak values of the apixaban concentration measured by LC–MS/MS or anti-Xa were observed between patients with and without bleeding, on A5 or on A2.5. The only clinical characteristic that was significantly different in all patients with bleeding compared to patients without bleeding was HAS-BLED score. Patients with a thromboembolic event had a lower average peak apixaban concentration, were older, and had higher HAS-BLED score. There was no difference in trough apixaban levels and the difference in CHA_2_DS_2_VASc score was of borderline significance. Neither trough nor peak average CVs were associated with bleeding or thromboembolic events (Table 3).

**Table 3:** Characteristics of patients with bleeding or thromboembolic event (TE) and patients without.

	With bleeding (N = 21)	Without bleeding (N = 41)	*p*	With TE (N = 4)	Without TE (N = 58)	*p*
Apixaban dose (5 mg/2.5 mg)	12/9	20/21	0.73	1/3	31/27	0.56
Age (years)	79 ± 6	77 ± 9	0.64	85 ± 3	77 ± 8	0.03
CHA_2_DS_2_VASc score	3.9 ± 1.3	4.1 ± 1.7	0.58	5.3 ± 0.5	3.9 ± 1.5	0.07
HAS-BLED score	1.3 ± 0.6	1.0 ± 0.5	0.03	1.8 ± 0.5	1.0 ± 0.5	0.03
LC–MS/MS trough (ng/mL)	96 (78–160)	101 (83–135)	0.98	91 (81–103)	103 (82–140)	0.44
CV trough (%)	18 ± 12	22 ± 16	0.37	22 ± 10	20 ± 15	0.56
LC–MS/MS peak (ng/mL)	221 (163–309)	211 (169–255)	0.54	153 (149–169)	219 (167–283)	0.05
CV peak (%)	19 ± 13	21 ± 10	0.43	25 ± 8	20 ± 11	0.23
Berichrom Heparin anti-Xa trough (ng/mL)	89 (76–152)	97 (78–119)	1.00	90 (78–99)	96 (77–133)	0.50
Berichrom Heparin anti-Xa peak (ng/mL)	184 (158–247)	180 (155–209)	0.43	152 (129–170)	184 (158–220)	0.09
STA–Liquid anti-Xa trough (ng/mL)	77 (63–121)	80 (67–98)	0.97	74 (63–81)	80 (64–111)	0.74
STA–Liquid anti-Xa peak (ng/mL)	192 (136–238)	180 (155–209)	0.19	131 (116–140)	170 (135–209)	0.09

Results are shown as number of cases, average ± SD or median with first to third quartile.

*CV* oefficient of variation.

## Discussion

Routine laboratory monitoring is not recommended for apixaban treatment in daily clinical practice. However, in some clinical situations, knowledge of on-treatment drug levels should be desirable for management strategies. There is a pronounced inter-individual variability, with up to sixfold variation in trough plasma apixaban concentrations^3,5,8,9^. In this study, we also examined intra-individual variability over time and the association of trough and peak apixaban concentration with clinical outcomes.

We obtained three trough and three peak samples approximately 6–8 weeks apart at a steady state of anticoagulant treatment for each patient with atrial fibrillation. The intra-individual variability in apixaban trough and peak concentrations measured by LC–MS/MS was relatively high and significant in patients on apixaban 5 mg twice- daily, but less pronounced in patients on apixaban 2.5 mg twice- daily. Patients with the highest trough apixaban variability (CV ≥ 30%) had lower body weight and creatinine concentration than other patients. They also achieved lower average trough apixaban levels. It may be speculated that these patients, on average 78 years old, had a lower volume of distribution, their renal function may be labile (dependent on hydration and diuretic use) and underestimated due to reduced muscle mass, and this may lead to fluctuation in apixaban clearance that varies over time. Other factors affecting the absorption of apixaban cannot be excluded.

Functional chromogenic anti-Xa assays, appropriately calibrated are rapid, simple to run, and are used for indirect apixaban determination. On contrary to intra-individual variability results in trough levels measured by LC–MS/MS, no differences were observed between three trough levels assessed by the two anti-Xa assays. It has been documented previously^11^ and confirmed by our study (Fig. 1) that anti-Xa measurements at low but clinically relevant levels (i.e. < 30 ng/mL) are less accurate. It appeared that apixaban concentrations measured with both anti-Xa assays were underestimated. This finding has already been reported for STA–Liquid anti-Xa^4^. The same study reported an overestimation of apixaban measured with the Berichrom Heparin assay, which is in contrast to our findings and could be attributed to different statistical calculations performed in both studies. Apixaban levels determined with anti-Xa are still informative in every day clinical practice despite lower accuracy as LC–MS/MS is not always (if at all) available.

The AVERROES substudy showed 20% lower trough median anti-Xa levels in patients who fulfilled criteria for the reduced dose of apixaban^4^. Our patients receiving 2.5 mg apixaban twice daily had 25–28% lower median trough concentrations (with all methods) compared with those receiving 5 mg twice daily. Our results are consistent with other studies in real-life patients, including patients on lower than the recommended dose, that showed even more than 30% differences in trough apixaban levels between patients on the high and the lowdose^5,8,9^. Moreover, we also showed a similar difference for peak apixaban levels between the two dosage groups. In our study, only 30% of patients met all the criteria, while the others had only one criterion for apixaban dose reduction. This reflects the prescriber's perception of increased bleeding risk in these patients. In daily clinical practice, many patients are treated with a reduced dose, outwith the label recommendations for this reduced dose treatment^12–16^. In the ORBIT-AF registry II (Outcomes Registry for Better Informed Treatment of Atrial Fibrillation phase II), apixaban was the most frequently underdosed direct oral anticoagulant^12^. In accordance with our results, old age was the most common reason for underdosing^12,17^.

Our previous studies have shown that real-life atrial fibrillation patients on the reduced dose of dabigatran or rivaroxaban had similar drug exposure than patients who received the full dose, although they did not meet all the criteria for a reduced dose^18,19^. When the dose is adjusted in patients on dabigatran or rivaroxaban, the full dose is reduced by only one-third, whereas it is reduced by one-half in patients on apixaban. Therefore, it appears that patients on apixaban may be at risk of an undesirably low trough concentration if the currently used clinical criteria for dose reduction are not strictly followed. Several authors have reported that underdosing was associated with a higher risk of stroke and all-cause mortality^13,14^. There is only one study that showed no increase in thromboembolic events after intentional underdosing according to the plasma peak concentrations in Japanese patients with atrial fibrillation^9^.

During our study, we observed 21 (34%) patients with bleeding (most of them with minor, self-reported bleeding at regular visits, only two with major bleeding) and 4 (6%) thromboembolic events (no ischaemic strokes, only one transient ischemic attack, and three acute myocardial infarctions). There was no association between bleeding events and through or peak apixaban levels. On the contrary, the AVERROES sub-study demonstrated a relationship between minor bleeding and anti-Xa levels^3^, but in our study the only bleeding predictors were clinical characteristics of the patients expressed by the HAS -BLED score. There were only 4 patients with a thromboembolic event and these had lower peak apixaban concentrations and were older than patients without such an event. Three patients who experienced an acute myocardial infarction were on the low dose of apixaban, although they met only one criterion for the reduced dose. However, they were at high risk for a thrombotic event due to known coronary artery disease. Santos et al. also reported more myocardial infarctions in under-dosed patients compared to patients on recommended dose. This supports the recommendation that in coronary patients with atrial fibrillation apixaban dose should not be reduced with concomitant antiplatelet therapy unless two or more criteria for dose-reduction are present^20–22^.

The main limitation of this study is a relatively small number of patients included. In addition, event rates were low, which limits the power to demonstrate an association between apixaban exposure and bleeding or thromboembolic events.

In conclusion, our study showed a high intra-individual and inter-individual variability in apixaban trough and peak concentrations in patients with atrial fibrillation. Neither variability nor trough apixaban concentrations were associated with clinical outcome. Furthermore, patients without criteria for the reduced apixaban dose may might be at risk for excessively low apixaban trough levels.

## Patients and methods

### Patients

We included 62 European ethnicity patients with atrial fibrillation treated with apixaban on average for 10 ± 7 months at our Anticoagulation Clinic (University Medical Centre, Ljubljana, Slovenia). There were 32 patients on apixaban 5 mg (A5) and 30 patients on apixaban 2.5 mg (A2.5) twice a day. A lower dose of apixaban was prescribed to patients with at least two of the following characteristics: age ≥ 80 years, body weight ≤ 60 kg, or serum creatinine ≥ 133 μmol/L. At the discretion of the treating physician, a lower dose of apixaban was prescribed also to frail patients and those with antiplatelet drugs. None of the patients received strong P-gp/CYP3A4 inhibitors or inducers. Only 3 patients used amiodaron, a moderate P-gp/CYP3A4 inhibitor. Demographics, thromboembolic and haemorrhagic risks according to the scoring system CHA_2_DS_2_VASc and HAS-BLED were recorded^23,24^. Renal function was estimated by the Cockcroft–Gault equation.

For each enrolled subject we collected three trough (trough 1, 2 and 3) and three peak (peak 1, 2 and 3) blood samples with an interval of 6–8 weeks apart. The trough concentration samples were collected 12 ± 1.5 h after the previous apixaban dose and the peak concentration samples were collected 123 ± 6 min after dosing. Patients reported that they had not missed any doses in the last week prior to the blood sampling. In one patient on A5 only one peak and one trough blood collection were obtained and one patient on A2.5 missed the third scheduled blood sampling. Follow-up was completed in August 2019 and the observation period was 30 ± 15 months. Follow-up visits at the clinic were scheduled once a year if there were no complications of treatment. Bleeding or thromboembolic events were documented regardless of when the event occurred in relation to the blood sampling. Major bleeding was defined according to the criteria of the International Society on Thrombosis and Haemostasis (ISTH)^25^. All other bleeding was considered minor. For patients that did not attend a scheduled appointment, inquiries were made with the designated person. During the observation period 2 patients died due to sepsis, in 6 patients treatment was permanently discontinued (1 due to major bleeding, 1 after successful ablation of atrial fibrillation, other due to frailty or renal failure), 4 patients were switched to warfarin and 4 to rivaroxaban.

All patients signed an informed consent form agreeing to participate in the study. The study was approved by the Medical Ethical Committee of the Slovenian Ministry of Health.

### Laboratory methods

Blood samples were drawn from the antecubital vein into 4.5 mL vacuum tubes containing 0.11 mol/L sodium citrate (9:1 v/v) (Becton Dickinson, Vacutaineer System Europe, Heidelberg, Germany). Plasma was prepared with 20-min centrifugation at 2000 × g, aliquoted into plastic vials, snap-frozen in liquid nitrogen and stored at − 70 °C until analysis.

Apixaban concentration was measured in plasma with LC–MS/MS as described earlier^5^. The lower limit of detection for LC–MS/MS was 2 ng/mL. Anticoagulation effects of apixaban were assessed with the following coagulation assays: activated partial thromboplastin time (APTT) (Pathromtin SL) and prothrombin time (PT) (Thromborel S), both Siemens Healthcare Diagnostics Products GmbH, Marburg, Germany on the CS2100i automated coagulation analyzer (Sysmex, Kobe, Japan); and with two anti-Xa assays: at University Medical Centre Ljubljana with the Berichrom Heparin on the CS-2500 coagulation analyzer (Sysmex, Kobe, Japan) and at Karolinska Institute with the STA–Liquid anti-Xa (Diagnostica Stago, Asnieres, France) on the CS2100i coagulation analyzer (Sysmex, Kobe, Japan). Both assays were calibrated with the STA-Apixaban calibrator (Diagnostica Stago, Asnières sur Seine, France). For Berichrom Heparin the original protocol provided by the manufacturer was slightly modified: the addition of exogenous antithrombin was left out.

All methods were carried out in accordance with relevant guidelines and regulations.

### Statistical methods

The within-subjects trough and peak coefficients of variation (CVs) were calculated as standard deviation/average × 100 (%) from all trough and all peak measurements of each individual. The average CV ± standard deviation was calculated as a measure of repeatability of trough and peak level. Differences between measurements on the three occasions were tested with the Friedman ANOVA. Differences between the groups of patients were tested with the Mann–Whitney U-test or with the χ^2^ test. Associations between variables were tested by the Spearman rank correlation coefficient. Statistical analysis was performed using Statistica Software (StatSoft, Texas, USA).
